# The challenge of cancer in middle-income countries with an ageing population: Mexico as a case study

**DOI:** 10.3332/ecancer.2015.536

**Published:** 2015-05-07

**Authors:** Ajay Aggarwal, Karla Unger-Saldaña, Grant Lewison, Richard Sullivan

**Affiliations:** 1 Institute of Cancer Policy, Kings College London, Guys Campus, Department of Research Oncology, Bermondsey Wing, London, SE1 9RT, UK; 2 Department of Clinical Oncology, Guys and St Thomas’ NHS Trust, London SE1 9RT, UK; 3 Mexican National Cancer Institute (INCan), 14080 México, D.F.; 4 International Prevention Research Institute (iPRI), Lyon 69006, France

**Keywords:** geriatric oncology, low middle-income country, demographic transition, ageing, cancer burden

## Abstract

Mexico is undergoing rapid population ageing as a result of its epidemiological transition. This study explores the interface between this rapid population ageing and the burden of cancer. The number of new cancer cases is expected to increase by nearly 75% by 2030 (107,000 additional cases per annum), with 60% of cases in the elderly (aged ≥ 65). A review of the literature was supplemented by a bibliometric analysis of Mexico’s cancer research output. Cancer incidence projections for selected sites were estimated with Globocan software. Data were obtained from recent national census, surveys, and cancer death registrations. The elderly, especially women and those living in rural areas, face high levels of poverty, have low rates of educational attainment, and many are not covered by health insurance schemes. Out of pocket payments and private health care usage remain high, despite the implementation of Seguro Popular that was designed to achieve financial protection for the lowest income groups. A number of cancers that predominate in elderly persons are not covered by the scheme and individuals face catastrophic expenditure in seeking treatment. There is limited research output in those cancer sites that have a high burden in the elderly Mexican population, especially research that focuses on outcomes. The elderly population in Mexico is vulnerable to the effects of the rising cancer burden and faces challenges in accessing high quality cancer care. Based on our evidence, we recommend that geriatric oncology should be an urgent public policy priority for Mexico.

## Introduction

In 2010, the Global Health and Ageing report estimated that 524 million people were aged ≥ 65, representing 8% of the world’s population. This figure is due to triple to 1.5 billion by 2050 (16% of the world’s population) [1] with 80% expected to be living outside of high-income countries [1]. (Classification of countries by income status has been performed according to World Bank criteria, based on Gross National Income per capita [2]. Worldbank. World Bank Atlas Method http://data.worldbank.org/about/country-classifications2013 [23rd August 2013]. Available from: http://data.worldbank.org/about/country-classifications.)

Relative to high-income countries, population ageing is occurring at a much faster pace in middle-income countries [3]. Between 2010 and 2030, the proportion of the population aged ≥ 65 in Brazil and China is expected to increase by 92% and 101%, respectively, compared to United Kingdom and United States, who are expected to experience a 30% and 49% increase in the same time period, albeit on a background of population ageing over several decades [4].

As a result, it is predicted that by 2030, approximately 70% of the anticipated 20 million new cancer cases worldwide will be in emerging economies, many of which lack the health care programmes required to manage their current cancer burden effectively [5, 6]. However, an area of public policy that remains under-represented in cancer control strategies in middle-income countries (MIC) is the impact of an increasing cancer burden on the expanding elderly population [7].

In 2011, 69% of cancers in men and 62% in women were diagnosed in those aged ≥ 65 in the United Kingdom [8]. Furthermore, relative survival of elderly patients with cancer is significantly worse compared to younger persons [9] with an ever-widening gap between elderly and middle–aged patients with cancer, considered to be due to poorer functional status, late diagnosis and inadequacy of treatment [10–14]. Similar trends have been observed in less developed countries, although individual country-level data remain limited [15].

Mexico is a MIC, ranked as the second largest economy in Latin America [2], with an estimated population of 117.4 million [16]. Like other emerging economies, it is facing up to the challenge of a rapidly ageing population. Currently, half of its resident population is 25 years or younger. However, the proportion of adults aged ≥ 65 increased from 4% in 1990 to 6.9% in 2012, an absolute increase of approximately 4.5 million people [17]. The current population aged ≥ 65 is 7.7 million and is expected to increase to 16.2 million people (11.5% of the population) by 2030, a projected 116% increase [17].

Mexico experiences a dual burden of disease akin to many MIC [18]. While 84% of deaths are due to non-communicable diseases of which 53% occur in individuals aged ≥ 65 [19, 20], ‘preventable causes’ such as infectious diseases, obstetric complications, and road traffic injuries are still important causes of death [21].

Disparities in the disease burden exist among the different states, attributed to variations in the extent of socioeconomic development [22]. The age-standardised death rates have been estimated to be 27% higher in the Southern Region (characterised by high levels of poverty) than in the Mexico City Metropolitan Area (MCMA), with the death rates from communicable diseases in this region approximately twice that of the Pacific Central Regions and MCMA [21].

The Mexican health system is characterised by fragmentation with several public purchasers vertically integrated with providers [19]. Social security services are available for the formally employed and their families. The largest is the Mexican Institute of Social Security (IMSS) [23]. IMSS is available for the employees of private enterprises and their families, and in 2012, it covered approximately 30% of the population [24]. An additional 7% are covered by other social security institutions, like ISSSTE (for employees of the State), PEMEX (for employees of the Mexican Oil Company), SEMAR (for Marines), SEDENA (for the military), and others [24]. For workers in the informal sector (self-employed persons or wage earners who do not make social security contributions) Seguro Popular (SP) provides cover through government sponsored facilities run by the Ministry of Health (MOH).

However, those unaffiliated have to pay out of pocket to attend Ministry of Health services or private health facilities, which provide a third of the hospital beds in the country. Over half of private facilities are located in Mexico City [25]. In general, affiliates to one scheme do not have access to others [26]. Mexico has among the lowest public spending on health care of OECD countries. In 2010, total health spending accounted for 6.3% of GDP of which 53% is financed from private expenditures [27]. Furthermore, according to an OECD study, the Mexican health system is among the least efficient. Reasons for this include inequitable health insurance coverage among lower income groups, unequal quality of services, high fragmentation in service provision, curtailment of patient choice, and lack of competition between providers. This is further exacerbated by weak regulatory powers of the MoH mainly due to the decentralisation of health services [26].

Overall, Mexico typifies the socioeconomic, epidemiological, and demographic changes experienced by other MIC. This study utilises several national-level data sources to explore the interface between rapid population ageing and cancer. Specifically, we include the projected cancer burden in the elderly, the prevalence of socioepidemiological factors that potentiate cancer risk and inferior outcomes, as well as a bibliometric evaluation of research output into cancer care in Mexico. We also explore the role of the Mexican health care system in widening or reducing health inequities. From the analysis, we set out proposals for future cancer and public policy research in Mexico and other MIC given the expected rise in cancer burden in this ageing population cohort.

## Methodology

### Literature review

A literature search was performed on 14 April 2015 using the following databases: PubMed, Medline, and Embase. Search terms were ‘cancer’ and ‘gerentology’, or ‘elderly’ or ‘older adult’, or ‘geriatric’ and ‘Mexico’, not New Mexico. Appropriate synonyms for cancer were utilised. Articles not available in English or Spanish were excluded. Additional searches were performed of ‘Mexico’ not New Mexico, plus one of the following terms: ‘cancer screening’, ‘chemotherapy’, ‘radiotherapy’, ‘geriatric oncology’, ‘social health insurance’, ‘catastrophic health expenditures’, and ‘out of pocket payments’.

### Analysis of Globocan database

In the absence of a national cancer registration system in Mexico (The ‘Registro Histopathalogico de Neoplasias Malignas’ (RNHM) was disbanded in 2004), accurate population cancer incidence is unavailable. The International Agency for Research on Cancer has compiled estimates of the worldwide incidence and mortality for 28 cancers in 184 countries [28]. Using this database, it was possible to estimate current and future (2030) cancer incidence in Mexico.

### Administrative data

A record of all age-specific cancer deaths in Mexico is available through the Directorate General of Health Information (DGIS). These data are compiled from death certificates by the Ministry of Health and the National Institute of Statistics and Geography (INEGI) [29]. The numbers of cancer deaths in Mexico were available from 1990 up to 2010.

### Bibliometric analysis

Bibliometrics is the quantitative analysis of research outputs and thus a validated, surrogate for overall research activity. Papers relevant to cancer research, from Mexico were identified in the Web of Science (WoS) database by means of a complex filter containing lists of specialist cancer journals and of title words indicative of cancer research, as previously described [30]. The bibliographic details of articles, notes, and reviews with an address in Mexico and published between 1989 and 2012, were downloaded to a series of files. Mexican cancer papers relevant to each of 16 cancer sites listed by the WHO in its Global Burden of Disease study, and four additional sites (brain, gallbladder, kidney, and testes) of importance for Mexico, were identified within the spreadsheet by means of a series of subfilters. The percentage of Mexican cancer research relevant to each site was then compared with the relative cancer burden estimated by GLOBOCAN, determined on the basis of both DALYs (Disability-Adjusted Life Years) and deaths. Similarly, the type of research (e.g., epidemiology, genetics, and surgery) was identified.

### Population estimates

Population estimates were provided by the National Population Council (CONAPO) for 2010–2050. This organisation develops yearly projections of population numbers by age, sex, and state. Projections are based on official national surveys and censuses which also reflect recent migration patterns [31].

### Population household surveys

Microdata from the *Censo de Poblacion y Vivienda* 2010 (National Housing and Population Census) were used to provide information on sources of income, employment status, levels of poverty, social security, and health services coverage of older persons [32]. A previous analysis by the National Population Council was used to guide this current review [33]. Key differences between male and female populations and rural and urban inhabitants were identified using pre-defined census criteria.

Microdata from the Study on Global Ageing and Adult Health (SAGE Mexico 2009–2010) were used to identify key sociodemographic characteristics in the elderly, including education status and marital status. The survey was developed by the WHO to ascertain patterns of health and well-being of adult populations in six low- and middle-income countries, including Mexico, China, and India [34].

Microdata from the National Survey on Nutrition and Health [35] were used to identify health service utilisation patterns, out of pocket health expenditures, prevalence of comorbidities, and functional and cognitive status of older persons.

## Results

### Patterns of cancer mortality in Mexico

**Figure 1.**
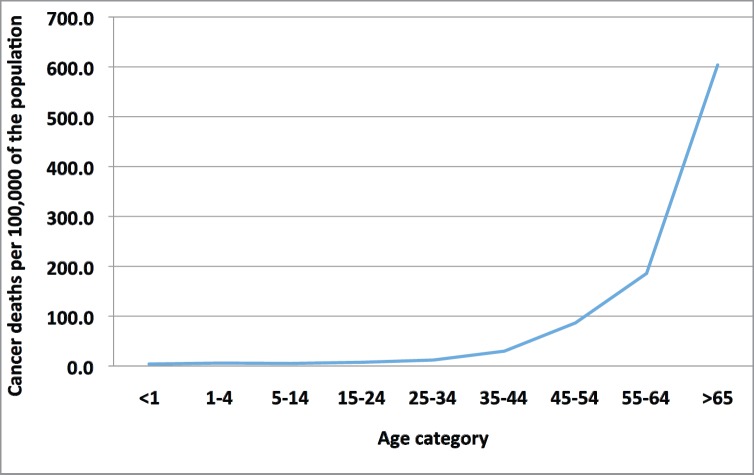
Number of cancer deaths (all tumour types) for both sexes per 100,000 of the population stratified by age category, 2010.

The rate of cancer deaths (defined as number of deaths per 100,000 of the population in that age category) increases sharply above the age of 45 (Figure 1). In 2010, 55% of cancer deaths occurred in men and women aged ≥ 65 despite this cohort representing only 6.2% of the total population. Additionally, the rate of cancer deaths was higher in men (721 per 100,000 of the population aged ≥ 65) compared to women (506 per 100,000 of the population ≥ 65).

In 2010, lung cancer (9.7%) caused the highest number of cancer-related deaths per annum (in all age groups) followed by gastric (8.0%) and prostate cancer (7.8%). Of note, 22.4% of deaths were due to malignancies for which the tumour site was not identified. Prostate (16%) and lung cancer (12.8%) are the predominant causes of cancer deaths in men at all ages. In women, breast (14%) and cervical cancer (11%) account for the highest proportion of cancer deaths.

In the population aged ≥ 65, prostate cancer was the most common cause of cancer death, accounting for 12.8% of all deaths, followed by lung cancer (11.9%), hepatocellular carcinoma (9.3%), and gastric cancer (8.4%). Breast and cervical cancer, although among the commonest causes of cancer death in women aged ≥ 65 (9.8% and 8.5% respectively), did not contribute a high proportion of deaths when considering the entire over-65 population (4.6% and 3.9%, respectively).

### Cancer incidence projections to 2030

Using Globocan 2012 software, we estimated that the effect of the demographic transition will result in an extra 107,000 cancer cases per year in Mexico by 2030 (a 72% increase compared to 2012). Globocan estimates predict that 58% of these cases will be in men and women ≥ 65, despite this group representing approximately 11.5% of the population [36].

We reviewed cancer site-specific incidence projections. There are expected to be an additional 7,900 new cases of lung cancer per annum by 2030 (8,600 cases in 2012), 75% of these new cases will affect individuals aged ≥ 65 years, with a greater proportion in males. For colorectal cancer, there are expected to be approximately 7,072 additional new cases per year by 2030 (80% increase based on 2012 estimates) of which 62% will be in men and women aged ≥ 65 [36].

The majority of new prostate cancer cases currently occur in elderly men (54% in men aged ≥ 65 in 2012). By 2030, there is expected to be a 188% increase in the number of new prostate cancer cases, 58% of which will affect men aged ≥ 65. Similar increases are expected for other cancers, namely gastric cancer, pancreatic cancer, and hepatocellular carcinoma, with a predominance in men and women aged ≥ 65.

In contrast to prostate cancer, the majority of new breast cancer cases in 2012 were in women < 65 years of age (78% of all new cases). By 2030, there are expected to be approximately 13,981 additional new cases per annum, 38% [36] of which will be in women aged ≥ 65 [36].

### Research activity

Our literature review of ageing and cancer in Mexico identified 521 papers. Thirteen were duplicates. The title/abstract of the remaining 508 articles was reviewed. Fifteen published articles [37–51] and two conferences abstracts [52, 53] reported on some aspect of the interface of ageing and cancer or geriatric oncology in Mexico. These included epidemiological studies, single-centre case series, assessment of treatment outcomes in elderly cohorts, as well as exploration of cancer screening practices in older adults.

The bibliometric analysis demonstrated that Mexican cancer research focused on three main cancer types in the period 1989–2012: cervix, breast, and leukaemia, with the lowest level of site-specific research in oesophageal, bladder, and testicular cancer. Research output into lung and prostate cancer ranked 10th and 12th respectively, both representing approximately 2% of total cancer research output in Mexico during this time period.

**Figure 2.**
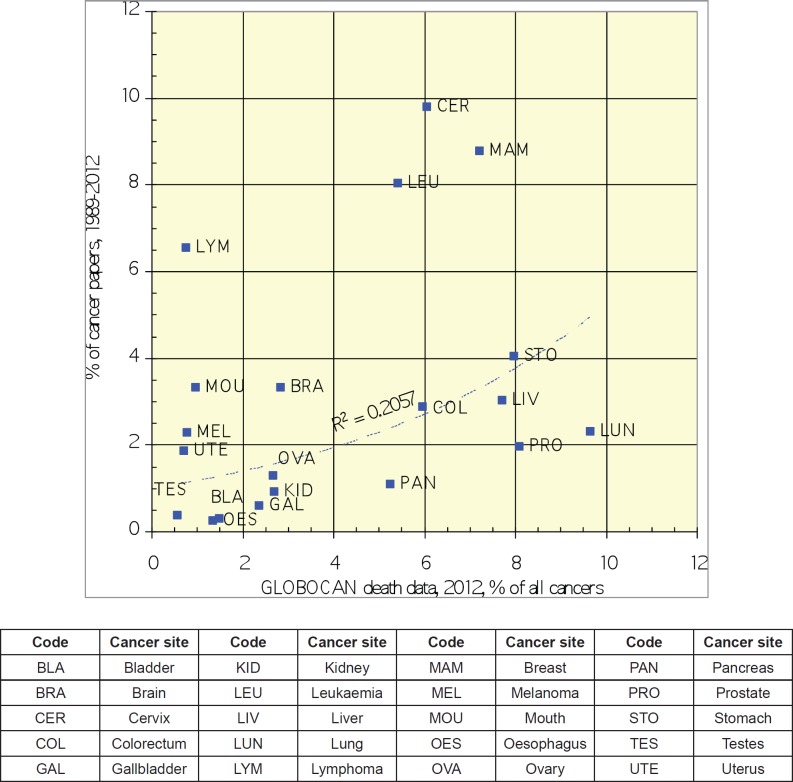
Graph depicting correlation between relative academic outputs from Mexico (1989–2012) for each cancer site versus disease burden for each cancer site (see key below graph).

**Figure 3.**
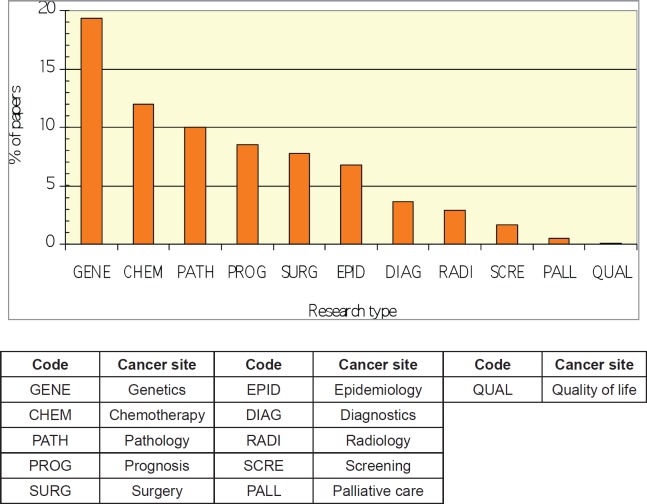
Graph demonstrating relative distribution of academic papers from Mexico, (1989–2012) stratified by research type (see key below graph).

There is a poor correlation between academic output for each cancer-type and cancer-specific mortality in Mexico (Figure 2). Relative to their disease burden (site-specific cancer mortality as a % of total cancer deaths) in the Mexican population as a whole, lung, pancreas, and prostate cancer do not have the commensurate representation in the research literature. In terms of research type, there is a heavy emphasis on genetics, with some effort on chemotherapy and pathology, but little on palliative care, quality of life, and disease epidemiology (Figure 3).

### Educational status

Analysis of the SAGE survey microdata demonstrated that nearly 50% of elderly urban residents (≥ 65) did not have any ‘formal education or did not complete primary school’ compared to 86.8% living in rural settings, (latest estimates show that 77% of men and women ≥ 65 are urban residents compared to 23% whose primary residence is in rural settings) [35]. Urban elderly residents were more likely to have completed higher education than their rural counterparts. There are no clear differences in educational attainment between elderly men and women.

### Employment status

Analysis of the Censo de Poblacion y Vivienda 2010 demonstrated that 64% of male rural residents, and 49% of male urban residents aged 65–69 continued to work. Nearly, 1 in 4 men (23%) aged ≥ 80 continued to work in rural settings compared to 1 in 7 living in urban areas. Although the proportion of women in urban and rural setting continuing to work ≥ 65 was lower than for men, elderly women play an important role in managing the household with over 50% of women in urban and rural settings aged ≥ 80 performing household activities.

### Sources of income and health insurance coverage

Financial contributions from family members within and outside of Mexico are an important source of income for elderly men and women (Table 1). In 2010, elderly men in both rural and urban settings received higher pension contributions than women. Furthermore, urban residents received higher pension payments than rural residents. Approximately, 80% of rural residents aged > 70 receive financial contributions from government programmes such as Oportunidades and Procampo.

In 2010, 30–40% of elderly rural residents did not have health insurance coverage compared to 20–25% of urban residents (Table 2). The oldest old (>80) were least likely to have insurance coverage, irrespective of residence. For elderly men and women in rural settings, Seguro Popular is the predominant insurer. In urban settings, IMSS is the main health insurer for the elderly.

### Duration of symptoms prior to seeking medical attention

Elderly men and women are more likely to experience medical symptoms for longer before seeking medical attention. Approximately, 35–40% men and women aged ≥ 65 had symptoms of greater than 12 months duration compared to 23% of those aged 20–64 (Table 3).

### Health care services utilised

**Figure 4.**
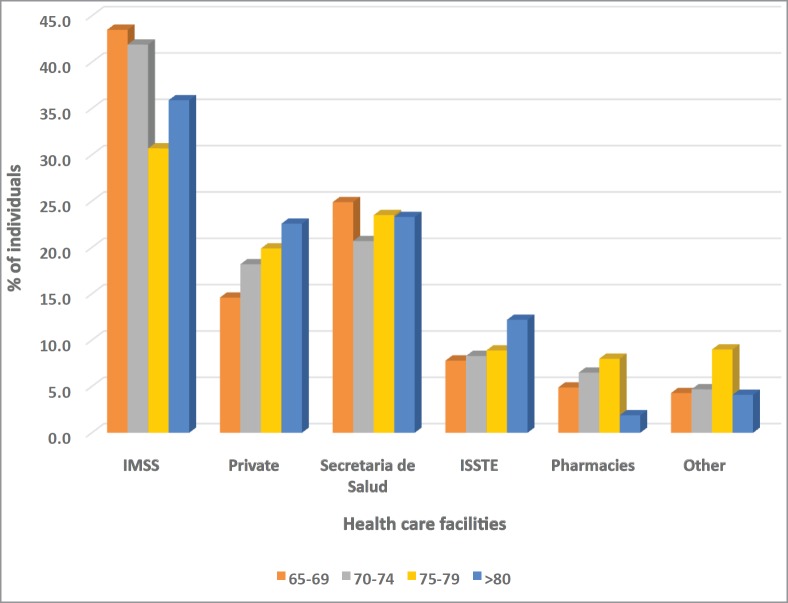
Graph of health care facilities utilised by respondents, stratified by age category. Other = Pemex/Defensa/Marina/IMSS Oportunidaes.

IMSS (social security sponsored facilities) and Ministry of Health (Secretaría de Salud) public facilities are most frequently utilised by elderly men and women. In addition, approximately, 25% of respondents aged ≥ 65 used private facilities when seeking medical attention (Figure 4)

### Out of pocket payments

Approximately, 25% of elderly men and women contribute financially towards the costs of their medical care. The mean expenditure was 210 Mexican Pesos (95% CI: 196–230) (equivalent to approximately 15 USD), and median 100 Pesos (7 USD). The amount paid was dependent on the health facility. Only 1% using IMSS facilities were required to pay compared to 14% using Ministry of Health facilities and 88% at private providers [35].

## Discussion

Globally, the cancer burden now falls hardest on emerging middle-income countries due to increasing exposure to pro cancer risk factors, as well as a rapidly ageing population. In Mexico, the proportion of the population aged ≥ 65 is expected to increase by 8.5 million by 2030. Our research demonstrates that 60% of newly diagnosed cancer cases will be in men and women ≥ 65 [36].

The majority of cancer deaths in Mexico occur in elderly men and women despite this cohort representing only 6.2% of the current total population [29]. While this reflects higher disease incidence in the elderly population, there is published evidence (albeit limited) of a survival differential between elderly cancer patients and younger adults in Mexico from certain cancers, including lung cancer [45], colorectal, gastric, [46–48], and breast cancers [38].

In Europe, this survival differential has been attributed to later stage of diagnosis, inadequacy of treatment, and barriers that the elderly face in accessing cancer services [55, 56]. In the absence of research evidence in Mexico, reasons for this trend among elderly cohorts can only be postulated from more generalised epidemiological cancer studies, the majority of which focus on breast and cervical cancer.

Socioeconomic factors account for higher rates of mortality from breast, cervical, and oropharyngeal cancers. These include poverty, lack of formal education, unemployment, rural residence, marginalisation (due to geographical isolation and transportation issues), and limited access to specialist oncological services [57–59].

Advanced stage at diagnosis has been highlighted as a key factor associated with worse survival outcomes in Mexico for breast and cervical cancer. Predisposing factors include low-screening uptake, lack of health insurance coverage, inability to pay for specialist services (diagnosis and treatment), and long waiting times for public services resulting in delays in diagnosis and referral for treatment [60–64] Our study therefore analysed the frequency of these risk factors in the elderly population from available national surveys.

The majority of elderly men and women in Mexico have had no formal education or only limited primary school education, with lower educational attainment in rural residents. Education is considered to affect health by ‘influencing knowledge and attitudes towards health as well as personal autonomy in decision making [66]. The Health, Well-being and Aging in Latin America and the Caribbean study (SABE) reviewed the association between education and cancer-screening practices among older adults (>60) in six cities across Latin America (including Mexico City). The study reported that illiterate or lower educated older men and women have the lowest rates of cancer screening compared with higher educated adults [49].

Difficulties in access to cancer services in rural settings have been well documented [68]. In 2012, 23.1% of men and women ≥ 65 were rural residents [24]. Elderly women are at greater risk of social isolation and marginalisation. Our analysis demonstrated that, approximately 50% of elderly women ≥ 65 were widowed compared to 15% of men [34]. In addition, households headed by women aged ≥ 65 were comparatively smaller than households headed by elderly men [32, 33]. This may have health implications with elderly female cancer patients shown to be at risk of under-treatment [56].

Poverty has the most dominant impact on health irrespective of age or gender. Studies over the last decade have shown that older Mexicans experience more poverty than working age cohorts, especially in rural settings [69]. The Household Income and Expenditure Survey (2008) demonstrated that amongst elderly populations (≥ 65), 14% are in food poverty, and 20% in capacity poverty (this includes non-monetary measures, such as education and health as well as basic food requirements) [70].

The low levels of social security support for the elderly in Mexico is a contributing factor with Mexico having the highest effective retirement age out of all OECD countries for men (72.2) and second highest for women (69.5) [71]. Without adequate coverage, they rely on their own capacity to work as an income source, or on the aid they can get from their families and/or government programmes (Table 2). About 64.8% of working older adults are employed in the informal sector and are therefore uninsured or have only Seguro Popular as health insurance compared with 48.4% of those working aged 15–59 [70].

This may prevent individuals from seeking medical attention in public settings due to the direct economic consequence of seeking care (lost earnings). The SABE study found that older women in Mexico city with no health insurance were less likely to have had a Pap smear as part of cervical cancer screening in the last two years compared with women with private or public health insurance [50]. This effect was independent of other socioeconomic indicators such as income, education, or occupation.

The decision to seek medical attention when new symptoms arise is an important factor in determining stage of presentation and likely survival [73]. Our analysis demonstrates that over 40% of older persons seeking medical attention experienced their presenting symptoms for greater than 12 months duration (Table 3). Delayed presentation could result in patients presenting with advanced staged disease, thus limiting their ability to receive curative or life prolonging treatment. In one retrospective study of 1019 primary lung cancer cases diagnosed between 1984–1992, the majority of patients were diagnosed with stage IIIb/IV disease [74]. The reasons remain unclear but may relate to issues of access, limited health literacy, [75] and poor functional status which has been observed in older Mexican adults suffering with chronic disease [40].

Fear of a cancer diagnosis, lack of knowledge or interest and anxiety regarding physical examination have all been cited as reasons for not attending cervical and mammographic screening appointments in the adult population [76, 77]. Elderly men are potentially vulnerable. Studies in Latin America investigating barriers to prostate cancer screening, highlight concerns about the impact of a digital rectal examination on their masculinity as well as an inherent stoicism towards their health, and an unwillingness to seek help [49]. Given that a number of malignancies potentially require invasive investigations such as colonoscopy, education is an important part of any policy relating to cancer in older persons, but this in itself requires greater research regarding perceptions of cancer and barriers to care amongst older persons.

IMSS is the largest health care insurer for elderly urban residents (65%). However, in rural localities, Seguro Popular dominates (55%); a reflection of poverty levels and the larger proportion who work in the informal sector [32, 78]. The uninsured population may be able to access cancer services through government sponsored facilities (MOH). However, eligibility is dependent on an individual’s income and patients still have to pay out of pocket (OOP) for any drugs [25]. Our analysis demonstrated 1 in 4 older persons reported paying OOP charges towards their health care, irrespective of health care provider.

Private providers remain popular with pharmacies in particular becoming an increasingly prominent health care provider [35]. An increased utilisation of private services has been reported among low- and middle-income groups covered by public insurance schemes, due to the fact that public services are often considered inferior amongst health users and may be difficult to access [78, 79]. There are concerns regarding excessive waiting times, particularly for diagnostic appointments due to excessive bureaucracy and case load. [25, 80, 81]. A study with 885 patients with breast cancer treated at two MoH hospitals, and two IMSS hospitals found a median time of five months between the first medical consultation and the beginning of treatment [63]. Many find that public sector health care facilities lack privacy, comfort or courtesy, and are concerned about a lack of quality of the treatment provided, with frequent misdiagnoses [58, 61, 68, 62]. Both insurance type and level of education have been shown to influence cervical and breast cancer screening in older Mexican women, with increased uptake among those in employment-based and private insurance schemes and higher education level [37, 42, 51].

Gaps in access also persist due to a lack of specialist human and organisational resources, particularly in remote rural areas. For example, there are 20 linear accelerators (delivering radiation therapy for cancer) for 32 states, with seven located in Mexico City [7]. These access gaps have been exacerbated since the introduction of Seguro Popular [83]. Despite a reduction in catastrophic health expenditures [84], there has been up to a 50% reduction in the availability of vital human and capital resources between 2008 and 2010, including hospital beds, doctors, scanning equipment, and pathology services [85].

In addition to SP, there is a Federal Fund for Protection against Catastrophic Health Expenditures (FPCHE) that covers high-cost health interventions for the uninsured [86]. This includes certain cancers affecting the adult population: breast, cervix, prostate, testicular, ovarian, colon, and non-Hodgkin Lymphoma. However, despite lung, gastric and hepatocellular carcinoma being three of the four most common causes of cancer death in Mexico, these are not covered by the scheme.

There are significant concerns. Firstly, these malignancies have no screening intervention in place which would facilitate earlier diagnosis. Secondly, the costs associated with diagnosis of these malignancies are potentially high. For instance, lung cancer requires complex imaging, multidisciplinary assessment and invasive biopsies [87]. Thirdly, survival outcomes for lung, gastric, and hepatocellular carcinoma remain poor even with maximal therapy.

As a result, elderly cancer sufferers not covered by social security face catastrophic and impoverishing expenditures and significant risk of morbidity and death from these malignancies. Given the poor prognosis from these malignancies if untreated or managed sub-optimally, palliative care services are highly important. However, surveys to date within Mexico highlight the difficulties in access to opioid analgesics for pain, the absence of palliative care policies and expertise, and the continued high usage of acute inpatient beds for patients dying with advanced malignancy, which uses valuable resources [88, 89]. One study reported that old age was associated with an increased likelihood of home death. This has implications for the organisation of palliative care services, with the necessity for strong community support [43].

Our review identified only a few studies exploring the impact of cancer in elderly populations. As described, the major causes of cancer death include lung, prostate, and gastric cancers, which have the highest incidence in elderly populations. However, our bibliometric analysis of research output from Mexico (1989–2012) reveals that despite the significant burden caused by these cancer sites, there is insufficient research reported in these cancer domains, which could have a direct impact on patient outcomes (Tables 2 and 3).

## Conclusions

### Cancer, ageing and research priorities in Mexico

In order to generate effective policy in this area, cancer in an ageing society needs to be considered an urgent research priority. This study has highlighted several future areas for research regarding ageing and cancer in Mexico to augment current understanding of the issues highlighted in this analysis:

- Cancer epidemiology in elderly populations.
- Cultural barriers, fears, and misconceptions of cancer in the elderly population.
- Health system barriers to effective cancer care in elderly populations
- Health literacy among elderly populations.
- Geriatric oncology and the assessment of elderly cancer patients for treatment.
- Treatment modalities utilised in elderly patients and their impact in terms of survival, toxicity and quality of life.
- Methods for improving cancer awareness in the elderly.

### Changing cancer public policy in Mexico

There has been increasing attention in the literature about the expected rise in cancer cases and the need for cancer control strategies to be implemented in Mexico and other Latin American countries [7, 90]. However, despite citing population ageing as the dominant factor in the expected rise in cancer cases, the impact of cancer on the ever-increasing elderly population remains neglected in the literature, particularly country-level outcomes.

This analysis highlights the paucity of evidence evaluating the interaction of older persons with cancer services in Mexico and the significant disparity in cancer research priorities given the expected increase in rates of lung, prostate and colorectal cancer. Elderly populations, particularly women and those living in rural areas, face significant barriers to accessing high quality cancer care and are vulnerable to diagnostic delays and potentially impoverishing expenditures. Seguro Popular was introduced to provide financial protection to these groups, however many of the cancers prevalent in older persons are not covered by the insurance scheme. This is an important public policy issue that needs to be addressed urgently as the impact on cancer outcomes and quality of life are potentially profound given the expected rise in cancer burden.

## Conflicts of interest

The author(s) declare that they have no conflict of interest.

## Figures and Tables

**Table 1.** Sources of income for elderly men and women (≥ 65) stratified by age category, sex, and place of residence, adapted from [33].

	Pension (%)		Help from relatives living outside of Mexico (%)		Help from relatives within Mexico (%)		Government programmes (%)		Other (%)	
Age Category	Male	Female	Male	Female	Male	Female	Male	Female	Male	Female
	**RURAL**										
65–69	16.1	6.4	6.7	8.5	4.9	8.3	33.7	43.4	1.7	1.4
70–74	20.8	7.5	7.1	8.7	5.7	9.2	70.2	74.8	1.4	1.4
75–79	17.7	8.2	8.6	9.2	8.1	10	82.4	85.4	1.6	1.5
80+	15.2	8.5	8.7	8	10.1	11.9	83.5	82.7	1.7	1.5
	**URBAN**										
65–69	46.5	24.3	2	3.6	4.7	10.3	6.7	10	2.7	3.9
70–74	54	27.8	2.8	3.8	6.8	13.1	23.1	26.8	2.9	3.7
75–79	55.5	29.7	4.1	4.2	9.2	15.3	29.1	33	3.9	4.2
80+	52.6	29.5	3.6	4.1	11.7	16.1	31.3	33	5	4.3

**Table 2.** Percentage of elderly men and women (≥ 65) covered by health insurance schemes, stratified by place of residence, sex and age category, adapted from [33].

	IMSS		ISSSTE		Seguro Popular		Other Public institution		Non-insured	
Age Category	Male	Female	Male	Female	Male	Female	Male	Female	Male	Female
	**RURAL**										
65–69	20.9	21.7	5.1	5.4	35.4	38.6	3.1	3.4	36.3	32.2
70–74	20.4	20.5	9.2	6.0	34.2	38.1	9.5	2.6	34.3	33.8
75–79	22.3	20.6	5.9	5.9	36.4	37.9	2.6	2.9	33.9	33.5
80+	19.7	17.4	5.5	5.4	34.3	35.5	3.1	2.8	38.5	39.9
	**URBAN**										
65–69	51.8	51.6	12.6	14.5	8.6	9.7	7.9	6.5	21.5	19.3
70–74	53.9	51.4	12.1	13.7	8.9	9.6	7.2	7.2	19.5	19.7
75–79	53.9	50.3	12.1	13.6	10.5	10.4	6.4	7.2	19.6	20.2
80+	52.9	46.2	12.6	14.4	9.5	9.8	6.3	7.6	21.0	24.3

**Table 3.** ‘Duration of symptoms prior to seeking medical attention for the last health problem the individual experienced’. Responses stratified by age category and sex.

	2 weeks (%)		2–4 weeks (%)		1–3 months (%)		3–6 months (%)		6–12 months (%)		>12 months (%)	
Age Category	Male	Female	Male	Female	Male	Female	Male	Female	Male	Female	Male	Female
20–64	49.0	45.6	11.2	11.8	8.1	8.3	4.0	4.6	3.0	6.9	24.6	22.2
65–69	36.4	35.6	8.2	11.7	5.3	3.9	5.0	5.4	2.1	4.2	42.9	38.4
70–74	36.4	32.2	6.8	7.7	3.6	5.0	4.4	8.9	6.4	5.5	41.8	40.6
75–79	39.3	31.2	7.3	9.3	2.5	10.0	0.0	5.9	7.2	9.1	41.5	34.0
80+	31.2	35.0	12.4	8.9	5.4	5.8	0.0	5.2	1.7	3.5	47.8	39.5
